# Quantitative Evaluation of Meniscal Healing Process of Degenerative Meniscus Lesions Treated with Hyaluronic Acid: A Clinical and MRI Study

**DOI:** 10.3390/jcm9072280

**Published:** 2020-07-17

**Authors:** Alessandra Berton, Umile Giuseppe Longo, Vincenzo Candela, Federico Greco, Francesca Maria Martina, Carlo Cosimo Quattrocchi, Vincenzo Denaro

**Affiliations:** 1 Orthopaedic and Trauma Surgery Unit, Campus Bio-Medico University, 00128 Rome, Italy; a.berton@unicampus.it (A.B.); v.candela@unicampus.it (V.C.); denaro@unicampus.it (V.D.); 2 Radiology Unit, Campus Bio-Medico University of Rome, 00128 Rome, Italy; federicogreco@outlook.com (F.G.); f.martina@unicampus.it (F.M.M.); c.quattrocchi@unicampus.it (C.C.Q.)

**Keywords:** degenerative meniscus lesion, conservative management, meniscal healing, T2 mapping, hyaluronic acid

## Abstract

Purpose: We aimed to evaluate clinical efficacy and healing effects of conservative management of degenerative meniscus lesions (DMLs) with a hyaluronic acid (HA) hydrogel. Methods: Patients were subjected to two HA injections two weeks apart. Western Ontario and McMaster Universities Osteoarthritis Index (WOMAC) and Patient’s Global Assessment (PtGA) and Clinical Observer Global Assessment (CoGA) of the disease were assessed at baseline, 30, and 60 days after treatment. Short Form (36) Health Survey (SF-36) was assessed at baseline and 60 days after treatment. One year after treatment, patients were called to know whether any of them had undergone arthroscopic partial meniscectomy (APM). All patients underwent magnetic resonance imaging using a 1.5-T Magnetic Resonance Imaging (MRI) scanner (Siemens Aera), which included a T2 mapping pulse sequence with multiple echoes at baseline and 60 days after treatment. Results: 40 patients were enrolled. WOMAC score, physical function subscale, PtGA and CoGA, and SF-36 showed a statistically significant difference between baseline and follow-up. One year after treatment, only one patient had undergone APM. A decrease in the T2 measurement was detected in the posterior horn medial meniscus in 39% of cases in both the red and red–white zone, and in 60% of cases in the white zone; in the posterior horn lateral meniscus in 55% of cases in both the red and white zones, and in 65% of cases in the red–white zone. Only for the latter, there was a statistically significant difference between baseline and posttreatment T2 measurements. Conclusion: This study supports the use of HA in the conservative management of DML as it is clinically effective and enhances meniscus healing as demonstrated by T2 measurements. Moreover, it reduces the need for APM at 1-year follow-up.

## 1. Introduction

Degenerative meniscus lesion (DML) presents in adult patients (35 to 65 years of age) who have not had a trauma and consists in a progressive delamination and surface fibrillation. DMLs cause knee problems for years, as they have been clearly correlated with the development of tibiofemoral osteoarthritis (OA) [1,2,3,4].

DMLs are completely different from traumatic meniscal lesions occurring in young patients. Traumatic meniscal lesions can be described as a true fracture produced by a twisting injury to the knee. Given the difference in morphological characteristics of DML and traumatic meniscal lesion, their management cannot be the same.

The management of DMLs deals with a contradiction between daily practice and scientific evidence [5,6,7]. Arthroscopic partial meniscectomy (APM) is frequently used for DMLs, which aims to relieve symptoms by removing torn meniscal fragments. The incidence of APM in the middle-aged has largely increased in several countries [8]. In the U.S., almost 700,000 APMs are carried out each year, with annual direct medical costs around $4 billion [9]. However, randomized controlled trials failed to demonstrate any additional benefit of APM for degenerative meniscal tears compared to non-operative treatment [10,11,12,13,14] or sham surgery [15,16]. All these studies except two [10,16] excluded patients with OA. In a multicenter, randomized, double-blind, sham-controlled trial, 146 patients with symptomatic DML and no knee OA were assigned to APM or sham surgery [15]. At 12 months after the procedure, there were no significant differences between groups in the Lysholm and Western Ontario Meniscal Evaluation Tool (WOMET) scores and in knee pain after exercise [15]. In a meta-analysis of nine randomized trials, the advantage of arthroscopic surgery compared with sham surgery, lavage, exercise, and medical treatment was small and limited in time [17]. Although well conducted, all the studies had biases and limitations. On the other hand, the cross-over from nonoperative treatment to arthroscopy ranged from 0 to 35%.

It should be taken into account that APM is associated with an increased risk of OA, as demonstrated by several studies. [18]. In a long-term follow-up study (mean 8.1 years), 57 patients who had undergone APM were clinically and radiographically evaluated for prevalence and progression of knee OA. Tibiofemoral OA was evident in 62.69%, and it progressed from 17.2% preoperatively to 65.95% postoperatively (*p* = 0.001) in the medial compartment and from 17.64% preoperatively to 58.82% postoperatively (*p* = 0.0324) in the lateral compartment.

The essential role of the meniscus in the lifespan of the knee has been clearly demonstrated in basic science and biomechanical studies [19]. The meniscus has multiple and complex functions, such as load-bearing, load transmission, shock absorption, stability of the knee, as well as lubrication and nutrition of articular cartilage [20]. The capacity to resist forces imposed on the knee is related to the biomechanical properties of the meniscal tissue. The meniscus is highly hydrated (72% water). The remaining content consists of extracellular matrix and cells. The organic matter is mainly constituted by collagen (75%), glycosaminoglycans (GAGs) (17%), DNA (2%), adhesion glycoproteins (<1%), and elastin (<1%) [20]. Age, injuries, and pathological conditions affect the proportion of biochemical contents [21,22]. The extracellular matrix and cells phenotype differ in the outer and inner portion. The outer portion is similar to fibrocartilage, while the inner portion has some characteristics similar to the articular cartilage. Cells of the outer portion are fibroblast-like cells, with an oval and fusiform shape. They are surrounded by type I collagen and small percentages of glycoproteins and type III and V collagen [23]. Cells of the inner portion can be considered as fibrochondrocytes or chondrocyte-like cells, with a round shape. They are surrounded mainly by type II collagen, a small amount of type I collagen, and a higher concentration of GAGs. Potential progenitor cells have been identified in the superficial zone. These cells are flattened and fusiform and have cell extensions [20].

In 2016, the European Society of Sports Traumatology, Knee Surgery & Arthroscopy (ESSKA) Meniscus Consensus analyzed literature and expert opinions to produce a guide for DMLs [5]. It stated that “surgery shouldn’t be proposed as a first line of treatment of DMLs (Grade A)”. Non-operative treatment can include rehabilitation, nonsteroidal anti-inflammatory drugs (NSAIDs), and intra-articular injections; however, there is “no evidence of which time/type of non-operative treatment should be proposed”.

Hyaluronic acid (HA) is a glycosaminoglycan that contributes to the viscoelasticity of the synovial fluid, and it is already used to provide additional shock absorption and prevent cartilage degeneration in OA [24]. Investigations have continued to reveal a variety of actions of HA, such as anti-inflammatory and analgesic functions [6,25]. It has been demonstrated that human synovial cells stimulated with HA increase the expression of Transforming Growth Factor beta 1 (TGF-β1) and Vascular-Endothelial Growth Factor (VEGF). Furthermore, in pathological human chondrocytes and synoviocytes stimulated by Interleukin 1 beta (IL-1β), HA was able to reduce the gene expression of degradative enzymes and inflammatory cytokines [26]. In inflammatory or degenerative joints, HA increased the synthesis of chondroitin sulfate and proteoglycans [27,28] and reduced the production and activity of Matrix Metallopeptidases (MMPs) and A Disintegrin and Metalloproteinase with Thrombospondin motifs (ADAMTS) [29,30,31].

The possibility of increasing levels of growth factors and counterbalance catabolic cytokine may open up new treatment option for patients with DMLs.

The potential role of HA in meniscal healing has been described in several in vivo studies [7,32,33]. Ishima et al. and Suzuki et al. found a significant increase in the healing rate of artificial tears injected with HA [32,33]. Ishima et al. [10] produced a longitudinal tear in the peripheral region of the medial meniscus and injected HA in the study group and saline solution in the control group. The HA group had a higher healing rate than the control group at 12 weeks.

Suzuki et al. [11] generated a cylindrical lesion on the lateral meniscus and injected HA in the study group and phosphate buffer in the control group. The HA group showed a significant increase in the rate of filling of the defect. The cell population of the repaired tissue shifted from fibroblast-like cells to chondrocyte-like cells. At six weeks, the ratio of chondrocyte-like cells to all cells was higher in the HA group, inducing authors to deduce that the healing rate was increased by HA.

Only recently, the underlying mechanism of HA in the meniscal healing process has been clarified. HA promoted human meniscus regeneration by inhibiting apoptosis, promoting cell migration, and accelerating cell proliferation, potentially through the phosphatidylinositol 3-kinase (PI3K)/mitogen-activated protein kinase (MAPK) pathway via the CD44 receptor [34]. Murakami et al. [34] analyzed the effects of HA on prostaglandin E2 (PGE2)-induced apoptosis and gene expression in meniscus cells. They showed that HA increases cell migration and proliferation in a concentration-dependent manner in both inner and outer meniscus cells. An anti-CD44 antibody blocked these effects. HA activated the phosphatidylinositol 3-kinase (PI3K) and mitogen-activated protein kinase (MAPK) pathways, and this effect was also blocked by an anti-CD44 antibody. Furthermore, HA upregulated the level of Collagen Type II Alpha 1 Chain (COL2A1) and ACAN mRNA of inner meniscus cells. Authors concluded that HA is expected to have clinical application in the management of meniscal injuries.

In the clinical setting, evidences about the efficacy of HA in the management of degenerative meniscus lesions are still scant. Only one prospective randomized trial on 50 subjects with DMLs, demonstrated that patients treated with a hydrogel based on a non-crosslinked HA alkylamide (HYADD4^®^; Fidia Farmaceutici SPA, Abano Terme, Italy) had a significant reduction in Visual Analogue Scale (VAS) pain and reduction in length and depth of the meniscal lesion [35]. However, further studies are needed to corroborate this result.

The challenge in studying the healing of the meniscus in humans is to identify a non-invasive and objective method to evaluate changes in meniscus composition. Conventional Magnetic Resonance Imaging (MRI) sequences are used to assess anatomy and detect morphological changes of the knee. Both radiography and morphologic MRI of the knee are not able to detect matrix alterations in DMLs and the response to infiltrative therapies. Using conventional MRI, measuring changes in meniscal tissue composition prior to surface breakdown is challenging. Several recent studies attest the role of quantitative MR imaging, such as T2 mapping, that is commonly used in the research field of knee OA [36,37]. T2 mapping is based on T2 relaxation time measurements and it has been extensively used to evaluate cartilage degeneration [38]. Recent studies have shown the potential of T2 relaxation time as biomarker to quantify meniscal degeneration in patients with knee OA [39]. Based on properties of biochemical tissue components, quantitative analysis of T2 relaxation times can reveal the composition of extracellular matrix, without the need of contrast or special MRI hardware. Increased T2 relaxation times indicate damage to the collagen network and a decrease in water content, both signals of tissue degeneration [40,41]. In vivo T2 mapping of the human meniscus correlates strongly with its degeneration, suggesting that T2 mapping enables detection and quantification of early compositional changes of the meniscus [40].

In the current preliminary study, we aimed to evaluate clinical efficacy and healing effects of conservative management of DMLs with a HA hydrogel Hymovis^®^ a sterile, non-pyrogenic, viscoelastic hydrogel for intra articular injection, Fidia Farmaceutici SPA). The primary aim of this study was to objectively demonstrate meniscal healing by T2 measurements, providing a quantitative evaluation of qualitative changes in the meniscus.

## 2. Materials and Methods

### 2.1. Study Design

The study is an open-label prospective pilot study. The study was approved by the local ethics committee (Ethics Committee Campus Bio-Medico University of Rome, Prot 19.17 OSS ComEt CBM). All patients signed informed consent before inclusion.

### 2.2. Participants

Patients were included in the study if they presented the following conditions: Male and female ≥ 35 and ≤65 years of age with degenerative meniscus lesions documented at MRI, without any history of significant acute trauma of the knee; no X-ray or MRI evidence of OA; patients disposed to observe requirements of the study for the whole time period.

Patients were excluded if they presented the following conditions: radiographic evidence of osteoarthritis in the target knee; knee ligament injuries; concurrent pathologies that would prevent the subject to proceed the study or that would interfere with the study results (e.g., rheumatoid arthritis, metabolic bone disease, gout, Paget’s disease, symptomatic chondrocalcinosis, etc.); recognized or presumed allergic reactions to hyaluronic formulations; recent operation to the knee in the 12 months before inclusion or planned surgery throughout the duration of the study; recognized or presumed infection of the joint; unsuitable skin status of the knee such as dermatitis or psoriasis; patient unable to undergo MRI for any reason; patient unable to follow the protocol for the entire length of the study.

### 2.3. Intervention

Patients were subjected to two Hymovis^®^ (HYADD4^®^, non-crosslinked HA alkylamide, 24 mg/3 mL, Fidia Farmaceutici SPA, Abano Terme, Italy), injections two weeks apart.

Hymovis^®^, highly purified sodium hyaluronate alkylamide obtained from bacterial fermentation, is a CE marked class III medical device, which is indicated for the treatment of pain in osteoarthritic joints and in the conservative treatment of the meniscal lesion of the knee and for the improvement of joint mobility through the enhancement of synovial fluid viscoelasticity.

Participants agree to interrupt NSAIDs consumption at least 24 h before each office visit for the entire study.

### 2.4. Evaluation of Clinical Efficacy

Clinical efficacy was assessed using the Western Ontario and McMaster Universities Osteoarthritis Index (WOMAC) questionnaire [42,43] and Patient’s Global Assessment (PtGA) and Clinical Observer Global Assessment (CoGA) of the disease. All patients were evaluated at baseline, 30, and 60 days after treatment.

The Short Form (36) Health Survey (SF-36 questionnaire [44,45] was collected at baseline and 60 days after treatment.

One year after treatment, patients were called to know whether any of them had undergone APM.

Local tolerability in the site of injection was assessed for redness and pain few minutes after each treatment session; local and systemic adverse events were registered for the duration of the study.

A diary of analgesic medications was required for the duration of the study.

### 2.5. Evaluation of Healing Effects

To objectively evaluate meniscal healing, a quantitative estimation of the meniscus was conducted using 1.5T MRI with T2 mapping technique at baseline and 60 days after treatment.

We obtained T2-mapping sequence in sagittal planes using a dedicated knee coil. A sagittal 2D multiecho spin-echo sequence with fat saturation was used with the following parameters: TR, 1500 ms; TE1: 10.9 ms; TE2: 21.8 ms; TE3: 32.7 ms; TE4: 43.6 ms; TE5: 54.5 ms; TE6: 65.4 ms; TE7: 76.3 ms; TE8: 87.2 ms; FOV read: 140 mm; FOV phase: 106.3; bandwidth: 372 hertz/pixel. MATRIX 340 × 320 mm; slice thickness 3 mm; distance factor 20% (0.6 mm). The acquisition time was 15 min 03 s.

Meniscal degeneration can be non-invasively quantified with T2 measurements [46]. How T2 relaxation works in the meniscus is not entirely clear. Available data refer to hyaline cartilage. There is a correlation between T2 relaxation and cartilage collagen orientation and water content [47]. Meniscal degeneration is characterized by matrix alterations such as degradation and disorientation of collagen interconnection, decrease of proteoglycans, enhanced water proportion, and penetration of synovial fluid into compromised locations [48]. Meniscal degeneration is represented by elevations in meniscal relaxation measures and it is correlated with clinical WOMAC scores [46]. Moreover, the value of T2 was proportional to the gravity of the meniscal degeneration [46]. T2 measurements offer an advantage over arthroscopy by examining the entire meniscus rather than the surface areas only.

**Figure 1:**
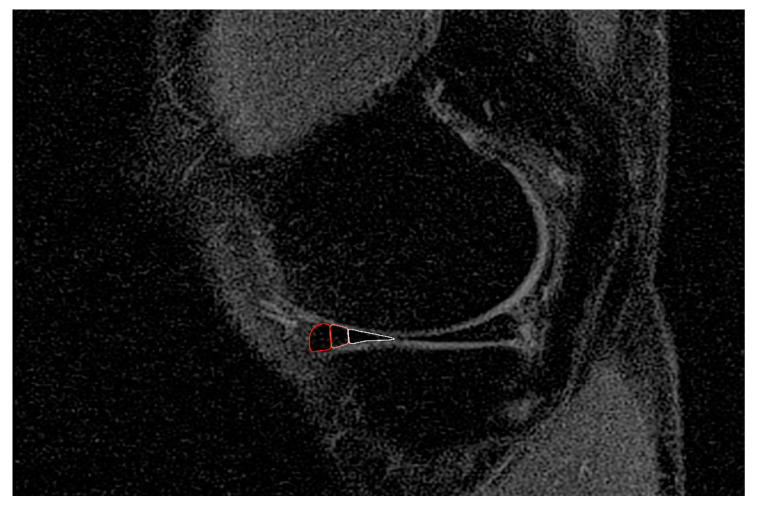
Red, red-white, and white zones of the meniscus.

The following meniscus compartments were analyzed: anterior horn lateral meniscus (AHLAT), anterior horn medial meniscus (AHMED), posterior horn lateral meniscus (PHLAT), and posterior horn medial meniscus (PHMED). The meniscal body was excluded because of partial volume effects. As the vascularity and cell profiling are different between the inner and outer meniscus, each compartment was divided into three zones: red, red-white, white (Figure 1).

An expert radiologist evaluated MRIs to define the degenerated meniscus compartment. Another radiologist performed T2 measurements in each zone of each compartment. Both radiologists were blind to patients’ data.

### 2.6. Correlation between Clinical Scores and T2 Measurements

The relationship between clinical evaluation and meniscal healing was assessed.

The Pearson’s correlation test was performed to correlate pretreatment (V1) clinical scores (WOMAC; WOMAC Section A, B, and C; PtGA; CoGA; and SF36) with pretreatment (V1) T2 measurements of each zone of each meniscus compartment. The same analysis was performed to correlate posttreatment (V3 and V4) clinical scores with posttreatment (V3 and V4) T2 measurements.

### 2.7. Data Analysis

A sample size of 40 participants was required to achieve statistical significance in the WOMAC score at a 0.05 level with 95% power.

Descriptive statistics were performed for each study time point for each variable collected: median, minimum, and maximum were determined for continuous variables and number and percentage of patients in each category for categorical data.

Non-parametric tests (Fisher’s and Wilcoxon test) were conducted to compare pre- and posttreatment data. *p*-values < 0.05 were considered statistically significant.

## 3. Results

Forty patients were enrolled in the study from July 2017 to September 2018 (M: 24, F: 16, mean age: 47 years, range: 35–65 years, mean male body weight 74 kg, mean male Body Mass Index (BMI) 25.1, mean female body weight 63 kg, mean female BMI 23.1). All patients completed the treatment, but one patient was lost at 60 days and another at 1-year follow-up.

### 3.1. Clinical Efficacy

**Figure 2:**
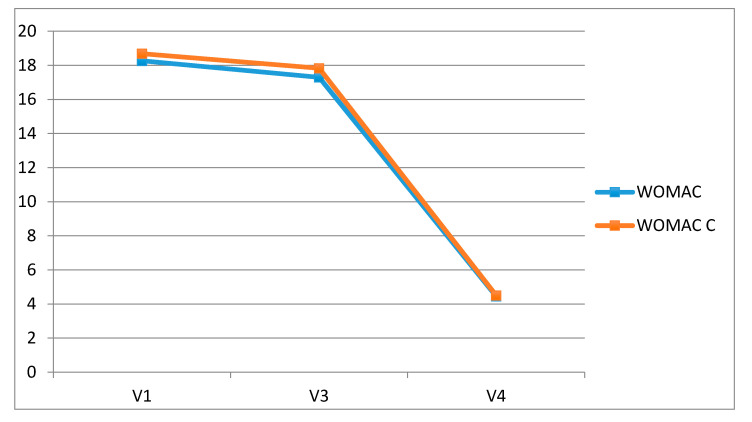
The Western Ontario and McMaster Universities Osteoarthritis Index (WOMAC) score and physical function subscale: 30 and 60 days follow-up.

The WOMAC score and physical function subscale (Section C) improved after treatment showing a statistically significant difference between baseline and 30 (*p* = 0.024 WOMAC, *p* = 0.04 WOMAC-C) and 60 days (*p* = 0.024 WOMAC, *p* = 0.02 WOMAC-C) follow-up (Figure 2).

**Figure 3:**
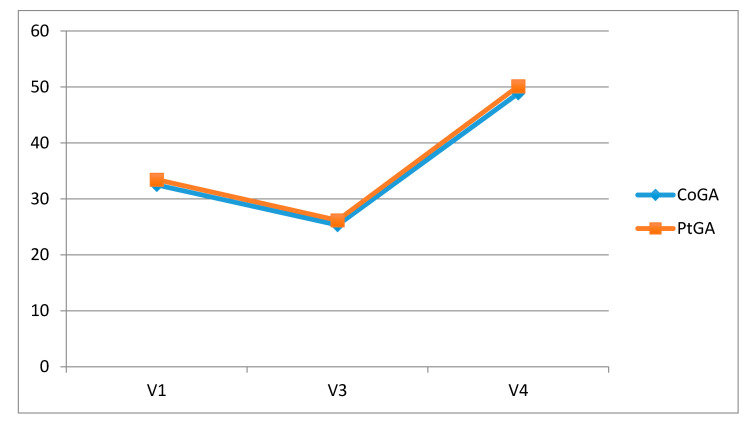
Statistically significant difference between baseline and 30 and 60 days follow-up of the Wilcoxon test. CoGA, Clinical Observer Global Assessment, PtGA, Patient’s Global Assessment.

PtGA and CoGA of the disease revealed an improvement over time. The Wilcoxon test showed a statistically significant difference between baseline and 30 (*p* = 0.008 PtGA, *p* < 0.001 CoGA) and 60 days (*p* = 0.001 PtGA, *p* < 0.001 CoGA) follow-up (Figure 3).

**Figure 4:**
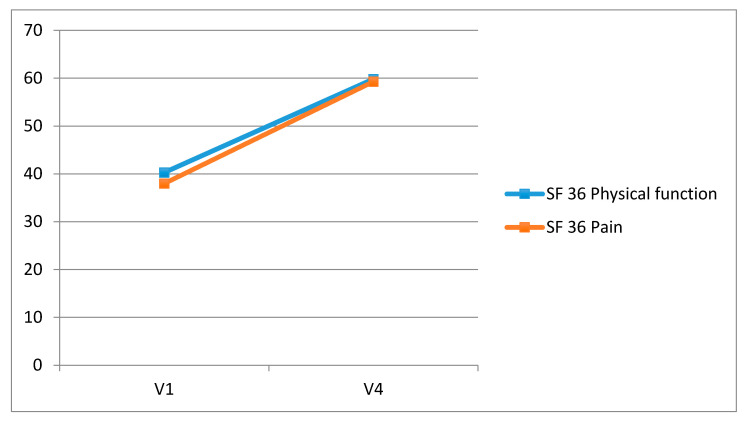
Statistically significant difference between baseline evaluation and 60 days follows-up of the Short Form (36) Health Survey (SF-36) physical functioning score and pain score.

The SF-36 physical functioning score and pain score showed an improvement with a statistically significant difference between baseline evaluation and 60 days follows-up (*p* = 0.01 SF-36 physical functioning, *p* = 0.03 SF-36 pain score) (Figure 4).

One year after treatment, only one patient had undergone APM. He had a BMI higher than the average of the patients.

Treatment showed good local tolerability, as no redness or pain was detected in the site of injection few minutes after each treatment session. No local or systemic adverse events were registered for the duration of the study.

Only 4 patients required analgesic medications from 3 to 10 days after the first or second injection (1 patient ibuprofen 800 mg × 5 days after the first injection, 1 patient ketoprofen 80 mg × 3 days after the first injection, 1 patient ketoprofen × 5 days) after the first injection; 1 patient paracetamol 1000 mg × 3 days after the second injection).

### 3.2. Healing Effects

**Figure 5:**
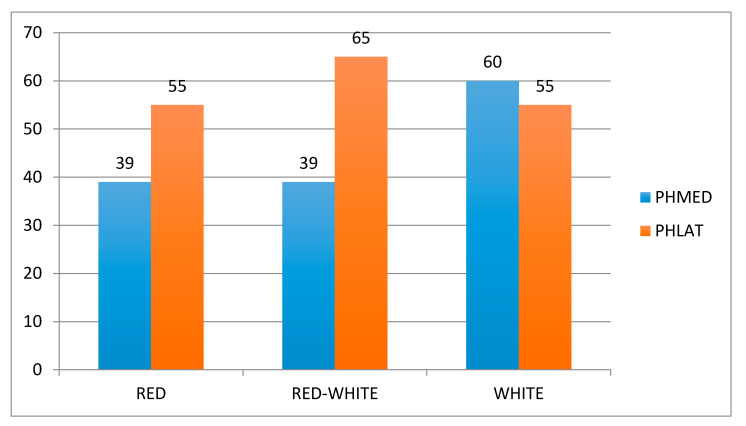
Statistically significant difference between baseline and posttreatment T2 measurements.

At MRI, the PHMED was degenerated in 33 cases, the PHLAT in 20 cases, the AHMED in 4 cases, and the AHLAT in 7 cases. MRIs of 4 patients were excluded because of artifacts that altered T2 measurements. Decrease in T2 measurement after treatment was detected in 13/33 (39%) cases in both the red and red–white zone of the PHMED, in 20/33 (60%) cases in the white zone of the PHMED, in 11/20 (55%) cases in both the red and white zone of the PHLAT, and in 13/20 (65%) cases in the red–white zone of the PHLAT. Only for the latter, there was a statistically significant difference between baseline and posttreatment T2 measurements (*p* = 0.03) (Figure 5).

### 3.3. Correlation between Clinical Scores and T2 Measurements

A statistically significant correlation was observed between pretreatment (V1) CoGA and all the zones of the AHMED (red zone *p* = 0.030, red and white zone *p* = 0.020, white zone *p* = 0.054), and between pretreatment (V1) PtGA and the red and white zone of the AHMED (*p* = 0.067). Pretreatment (V1) SF36 mental health score was significantly correlated with the red zone (*p* = 0.036) and red and white zone (*p* = 0.018) of the PHMED; SF36 physical functioning score was significantly correlated with the white zone of the PHMED (*p* = 0.048).

Posttreatment (V4) SF36 emotional role functioning was significantly correlated with the white zone of the AHLAT (*p* = 0.047), and SF36 physical functioning score was significantly correlated with the red zone of the PHLAT (*p* = 0.038).

## 4. Discussion

The most important finding of this preliminary study is that HA is a valuable biologic solution for meniscal healing. It demonstrates clinical efficacy and healing effects of conservative management of DMLs with a HA derivative hydrogel (Hymovis^®^, HYADD4^®^, Fidia Farmaceutici SPA). The treatment of DMLs with HA showed a marked improvement in patient-reported outcomes that was maintained 60 days after treatment. For the first time, the healing effect of HA on DMLs has been objectively demonstrated, thanks to T2 mapping technique. A high percentage of cases showed a decrease in meniscal relaxation measures, with statistically significant differences in the red–white zone of the PHLAT. This finding can be related with improvements in macromolecular structure of collagens, proteoglycan, and water.

The recent knowledge about the effects of HA on proliferation and migration of human meniscus cells, and the underlying healing mechanisms, can explain the result of this study. Murakami et al. [34] demonstrated that HA increases cell migration and proliferation in both inner and outer meniscus cells in a concentration-dependent manner. Meniscus cells from the outer and inner menisci were collected from 18 lateral menisci of patients who underwent total knee arthroplasty and were treated with HA or chondroitin sulfate. Prostaglandin E2 (PGE2)-induced apoptosis and gene expression were evaluated. Cell proliferation was induced by activating the CD44 receptor and PI3K and MAPK signaling. Moreover, HA also inhibited PGE2-induced apoptosis and increased the expression of extracellular matrix elements, particularly type II collagen and aggrecan. This effect was notably evident in inner avascular meniscus cells, whose profiling is similar to those of chondrocytes. Chondroitin sulfate downregulated MMP13 mRNA of both inner and outer meniscus cells, suggesting that chondroitin sulfate suppresses the inflammatory reaction rather than providing meniscal restoration.

The rationale of using HA on DMLs is sustained by some in vitro studies. Two preclinical studies have been conducted in rabbits with meniscal lesions [10,11]. Ishima et al. [32] created a longitudinal tear in the peripheral region of the medial meniscus. The target joint received HA injection once a week for 5 weeks (HA group), while the contralateral knee received saline injection (control group). Assessment was conducted 6 and 12 weeks after surgery. Meniscal healing was evident in both groups on gross morphological examination, but the HA group showed a significantly higher healing rate than the control group at 12 weeks. Meniscal healing on histological evaluation moved from the tibial portion in both groups at 6 weeks and proceeded toward the femoral surface at 12 weeks in the HA group. Suzuki et al. [33] produced a cylindrical lesion on the lateral meniscus and injected the knee with HA once a week. The meniscus was compared with control, injected with phosphate buffer, at 1 and 6 weeks. A significant increase in the rate of filling of the defect was detected in the HA group. As the repair progressed, the cell population of the repaired tissue shifted from fibroblast-like cells to chondrocyte-like cells. At six weeks, the ratio of chondrocyte-like cells to all cells was higher in the HA group, inducing authors to deduce that the healing rate was increased by HA.

In humans, the meniscus’ potential to heal is greater and depends on the location, type, length, and stability of the tear. In a retrospective evaluation of 3612 arthroscopic procedures, Weiss et al. [49] selected 80 stable meniscal tears (in 75 patients). At an average of 26 months, a new arthroscopy was performed on 32 patients (26 of whom had a longitudinal tear and 6 of whom had a radial tear). Complete healing was observed in 17 of the 26 longitudinal tears.

Our findings agree with previous studies. In a prospective randomized clinical trial Zorzi et al. [35] evaluated the efficacy of HA in the management of fifty subjects with DMLs. Patients were clinically evaluated at baseline and after 14, 30, and 60 days to assess pain (Visual Analog Scale), knee functionality (WOMAC questionnaire), and health status (SF-36). By comparing the follow-up MR images with the baseline images, the presence or absence of a reduction in length and depth of the lesion was identified. Authors found a significant reduction in VAS pain and reduction in length and depth of the meniscal lesion after treatment. In our study, we used an objective method to evaluate meniscal degeneration and the healing effects of HA. T2 measurements allow to quantify meniscal composition because of the correlation between T2 relaxation and biochemical tissue components [47]. Increased T2 relaxation times indicate damage to the collagen network and a decrease in water content [40,41].

T2 measurements are non-invasive and offer an advantage over arthroscopy by examining the entire meniscus rather than the surface areas only. T2 mapping provides a useful tool for clinicians in radiological analysis of the meniscus. The value of standard radiography is extremely limited for the assessment of meniscal pathology; it may be indicated to obtain a differential diagnosis and to estimate the grade of OA. Ultrasound is rarely used as a diagnostic tool for meniscal pathologies, and its accuracy is operator dependent. CT arthrography with multiplanar reconstructions can detect meniscus tears that are not visible on MRI, but it is an invasive technique. MRI is the most accurate and less invasive method for diagnosing meniscal lesions. MRI allows detecting and characterizing the meniscal lesion. Quantitative T2 and structural assessment of the meniscus with T2 mapping show significant correlations with the reference standard and is a useful technic to quantitatively evaluate qualitative changes of the meniscus after treatment. T2 measurements are sensitive to the content of water and concentration of macromolecules in the extracellular matrix and to interaction between biochemical components that is related with the content, orientation, and anisotropy of the collagen [50,51].

One of the limits of T2 mapping is the susceptibility to the magnetic angle effect. T2 values may be artificially elevated in specific regions depending on reciprocal orientation of meniscal components and the main magnetic field. Some studies suggest that T2 values are greater when the region of interest is oriented at 55° to B0 and lower in regions oriented at 180° (0°) to B0 [52]. We tried to reduce this effect using the same positioning in relation to the magnet for all patients.

When interpreting T2 mapping results, it is important to remember that the T2 value changes according to the strength of the magnetic field B0. Lower T2 values were observed at higher field strengths. In addition, sequence type, coil architecture, and calculation method of T2 mapping affect T2 results. A larger population and histological correlations with meniscal anatomic structure are needed to assess this.

This study shows a statistically significant correlation between some clinical scores and some T2 measurements in different zones of the meniscus. This finding can be interpreted as an inconstant relationship between clinical symptoms and degenerative characteristics of the meniscus that is already known. It is well established that DMLs are often incidental findings at MRI, and it is difficult to understand whether pain is directly produced by DML even if the lesion is unstable [53]. Incidentally, the progression of symptoms of DMLs is not clear. Data should have been stratified according to the degree of meniscal degeneration and patients’ BMI because of potential influence on the outcomes. Further studies are needed to investigate this aspect.

In this study, one year after treatment, none except one patient needed APM. In other Level I studies, the need of arthroscopy after conservative treatment without HA knee infiltrations was reported to be over 30%, and it was required at an interval of 3 to 6 months [54].

Findings of this study should be considered as preliminary because of the small study group. Results should be interpreted carefully, and larger confirmatory studies are needed.

The limitations of this pilot study include the absence of a control group because ethical issues preclude preforming placebo intra-articular injections. As an alternative to the control group, we can refer to the history of symptomatic DMLs treated with conventional conservative therapy. Conservative treatment of patients with mechanical symptoms is associated with poor outcomes, and over 30% of patients need arthroscopy at the second stage [54].

Another limitation of this study is the omission of evaluating treatment outcomes in proportion to the degree of meniscal degeneration. The healing effect of HA can be proportional to the degree of meniscal degeneration. A high degree of meniscal degeneration in enrolled patients could explain the results. It will be the subject of future endeavors.

The results of our study refer only to patients with symptomatic DMLs. We did not consider traumatic meniscal tears for the following reasons. Traumatic meniscal lesions are generally associated with acute onset of pain, swelling, catching, and locking of the knee. Patients affected by traumatic meniscal tears have usually a poor compliance to conservative treatment because of pain intensity and activity restriction. Those patients need to be surgically treated in a short time to relieve pain and regain physical function. In the clinical setting, there is no evidence about the efficacy of HA injections for traumatic meniscal lesions, representing an ethical issue.

In future studies, we aim to increase the study population to obtain more solid data to highlight the healing effect of HA and sustain the conservative treatment of DMLs with HA. Patients will be stratified based on the degree of meniscal degeneration and BMI. It is plausible that an early degenerative change may respond better to treatment.

## 5. Conclusions

This study supports the use of HA in the conservative management of DML as it is clinically effective and enhances meniscus healing as demonstrated by T2 measurements. Moreover, it reduces the need for APM at 1-year follow-up, representing a less invasive and cost-effective option compared to APM.

## Abbreviations

Degenerative meniscus lesion (DML), hyaluronic acid (HA), arthroscopic partial meniscectomy (APM), Patient’s Global Assessment (PtGA), Clinical Observer Global Assessment (CoGA), anterior horn lateral meniscus (AHLAT), anterior horn medial meniscus (AHMED), posterior horn lateral meniscus (PHLAT), and posterior horn medial meniscus (PHMED).
